# Honey as a Natural Antimicrobial

**DOI:** 10.3390/antibiotics14030255

**Published:** 2025-03-01

**Authors:** Matthew Chidozie Ogwu, Sylvester Chibueze Izah

**Affiliations:** 1 Goodnight Family Department of Sustainable Development, Appalachian State University, 212 Living Learning Center, 305 Bodenheimer Drive, Boone, NC 28608, USA; 2 Department of Community Medicine, Faculty of Clinical Sciences, Bayelsa Medical University, Yenagoa 569101, Nigeria

**Keywords:** honey, antimicrobial properties, antibiotic resistance, wound healing, natural remedies

## Abstract

Honey, a natural product with a rich history of medicinal use, has gained increasing recognition for its potent antimicrobial properties, particularly against antibiotic-resistant pathogens. This review focuses on the antimicrobial mechanisms of honey, including its efficacy against resistant bacteria, such as Methicillin-resistant *Staphylococcus aureus* and *Pseudomonas aeruginosa*. The antimicrobial action of honey is multifactorial, involving hydrogen peroxide production, phenolic compounds, high sugar concentrations, and the presence of bee defensin-1. The composition of honey varies based on its floral source, which can influence its antimicrobial strength. Certain types, such as Manuka honey, are particularly effective in clinical applications due to their higher levels of bioactive compounds. Honey has also been shown to disrupt bacterial biofilms, a major factor in antibiotic resistance, enhancing its therapeutic potential in treating chronic wounds and infections, especially in patients with compromised immune systems. Moreover, honey’s ability to improve wound healing, reduce inflammation, and promote tissue regeneration highlights its broad therapeutic profile. As antibiotic resistance continues to challenge modern healthcare, honey offers a promising complementary treatment in antimicrobial therapy. Research into its specific bioactive components and potential synergistic effects with other natural agents, like ginger and propolis, could expand its applications. Standardizing honey products for medical use and establishing clinical guidelines are essential for optimizing its therapeutic benefits. As scientific understanding of honey’s antimicrobial mechanisms deepens, its integration into healthcare systems as an adjunct therapy is expected to increase, offering a natural and effective alternative in the fight against infectious diseases.

## 1. Introduction

Honey has long been recognized for its natural antimicrobial properties, attributed to its low pH, high sugar content, and the presence of hydrogen peroxide and bioactive compounds such as flavonoids. Beyond honey, a variety of botanicals [1,2,3,4], including garlic, ginger, and turmeric; *Citrus* species [5,6]; *Vernonia* species, and turkey berry [7,8], possess antimicrobial properties. The antimicrobial potentials of plants is attributed to some bioactive compounds and essential oils that they contain [9,10,11]. Honey, a natural product produced by honeybees from the nectar of flowers, has been utilized for its nutritional and medicinal properties for centuries. It is a complex mixture containing approximately 80–85% carbohydrates, primarily fructose and glucose, along with water, vitamins, minerals, and various bioactive compounds such as phenolic acids and flavonoids [12]. The unique composition of honey not only contributes to its sweet flavor but also underpins its therapeutic potential, particularly in wound care and as an antimicrobial agent. The historical use of honey in various cultures, including ancient Egypt and Greece, highlights its significance as a natural remedy, where it was employed for treating wounds and gastrointestinal disorders [13,14].

The importance of antimicrobial agents in modern healthcare cannot be overstated, especially in the context of rising antibiotic resistance. Antimicrobial agents prevent infections, particularly in surgical settings and wound care. The emergence of multidrug-resistant bacteria has necessitated the exploration of alternative treatments, including natural products like honey, which exhibit potent antibacterial properties against a range of pathogens, including methicillin-resistant *Staphylococcus aureus* (MRSA) and *Pseudomonas aeruginosa* [14,15]. The ability of honey to enhance wound healing while combating infection makes it a valuable adjunct in modern medical practices, particularly in managing chronic and acute wounds [16,17]. Honey’s unique properties, particularly its antimicrobial and wound-healing capabilities, have been the subject of extensive research. The antibacterial activity of honey is attributed to several factors, including its high sugar content, low water activity, and hydrogen peroxide production [18,19]. Honey is a natural product with diverse types, including clover, acacia, manuka, and wildflower, each characterized by its unique floral origin and distinct metabolic composition. These variations contribute to its nutritional and therapeutic potential, as honey contains sugars, enzymes, amino acids, phenolic compounds, and antioxidants. Different types of honey exhibit varying levels of antimicrobial efficacy, influenced by their floral source and geographical origin. For instance, Manuka honey, derived from the *Leptospermum scoparium* plant, has gained prominence due to its high Unique Manuka Factor (UMF), which correlates with its antibacterial potency [20]. Studies have demonstrated that Manuka honey inhibits bacterial growth and synergizes with conventional antibiotics, enhancing their effectiveness against resistant strains [16,20].

Historically, honey has been used in various medicinal applications, ranging from wound dressings to treatments for respiratory ailments. Its application in wound care is particularly noteworthy, as honey has been shown to promote wound healing through multiple mechanisms, including inflammation reduction, tissue regeneration stimulation, and infection prevention [21,22]. Clinical studies have confirmed that honey dressings can accelerate wound healing and improve outcomes in patients with diabetic ulcers and burns [17,23]. The use of medical-grade honey, such as Medihoney™, has been endorsed by healthcare professionals as a viable alternative to conventional wound care products, particularly in cases where antibiotic resistance poses a challenge [16,21]. Manuka honey is rich in methylglyoxal and is known for its potent antimicrobial properties, while acacia honey is valued for its high fructose content and mild taste. The metabolic diversity of honey supports health benefits such as wound healing, immune modulation, and antioxidant protection [24]. This diversity highlights honey’s role as both a nutritional and medicinal resource.

The biochemical composition of honey, including its rich array of antioxidants, contributes to its health benefits. Phenolic compounds in honey have been shown to exert antioxidant effects, mitigating oxidative stress and inflammation and further supporting the healing process [25,26]. These bioactive compounds enhance honey’s antimicrobial properties and provide additional therapeutic benefits, such as anti-inflammatory and immunomodulatory effects [14,27]. This multifaceted action makes honey an attractive option in integrative medicine, where natural products are increasingly recognized for their potential to complement conventional therapies. Research should continue to explore the diverse applications of honey in modern medicine, particularly in the context of its antimicrobial properties. The growing body of evidence supports using honey as a topical agent for various skin conditions, including atopic dermatitis and skin ulcers [16,20]. Additionally, honey’s potential role in oncology is being investigated, with studies suggesting that the diverse physicochemical characteristics contributes to the chemoprotective properties, aiding in the prevention and treatment of cancer [27,28]. The versatility of honey as a natural product underscores its importance as a food source and a valuable therapeutic agent in contemporary healthcare.

This review explores honey’s antimicrobial properties, natural composition, and mechanisms that contribute to its effectiveness against various pathogens. It reviews the scientific evidence supporting honey’s role in healthcare, particularly in wound healing, gastrointestinal treatments, and combating antibiotic-resistant bacteria. The review highlights the diverse types of honey, such as Manuka honey, and their specific antimicrobial strengths. It also discusses the potential for integrating honey-based products into public health strategies, especially in low-resource settings, while addressing quality control and standardization challenges.

## 2. Types of Honey and Their Geographic Origins and Major Uses

The composition and properties of honey are significantly influenced by its geographical and botanical origins, which in turn affect its quality, nutritional value, and potential health benefits. Table 1 presents the various types of honey, detailing their primary floral sources, geographic regions, distinct characteristics, and primary uses. From the medicinal properties of Manuka honey to the culinary versatility of wildflower honey, each type offers unique benefits that cater to health, gastronomic, and therapeutic needs. The global spectrum of honey types reflects their adaptability and cultural significance, making them essential in traditional and modern lifestyles.

Geographical origin plays a crucial role in determining the physicochemical properties of honey. A study by Tomczyk et al. [27] demonstrated that honey samples from different locations exhibited significant variations in their physicochemical parameters, including mineral composition and antioxidant activity, underscoring the impact of geographical factors on honey quality. Similarly, Fangio et al. [28] noted that honey from the Miramar region of Argentina showed high inhibitory or antimicrobial efficiency on *Escherichia coli*. This suggests that geographical factors may indirectly influence honey’s properties through variations in botanical sources. Furthermore, Nyarko’s [29] research highlighted distinct physicochemical properties in honey from various regions in the United States, attributing these differences to geographical and processing factors. The influence of environmental conditions on honey characteristics is also well-documented. Zarei et al. [30] emphasized that moisture content, a critical quality parameter, is affected by climatic conditions during honey production, which varies by region. This is corroborated by findings from Alshareef et al. [31], who reported significant differences in pH levels among honey from different areas of Saudi Arabia, further illustrating how geographical factors can influence honey’s chemical properties. Additionally, Wu et al. [32] discussed how stable carbon isotopic compositions can reflect the environmental conditions of honey production, thereby providing insights into its geographical origins.

Botanical origin is another critical factor that interacts with geographical influences to shape honey’s characteristics. The study by Anyrjani [33] found that honey of the exact floral origin but from different geographical locations exhibited significant differences in diastase activity, indicating that both floral and geographical origins must be considered when assessing honey quality. This is supported by research from García-Tenesaca et al. [34], which highlighted the protective effects of monofloral honey against oxidative damage, demonstrating that botanical origin significantly contributes to honey’s health benefits. Moreover, Zhou et al. [35] utilized carbon isotope ratios to determine honey’s authenticity and geographic origin, emphasizing the importance of botanical and geographical factors in honey classification. The complexity of honey’s composition is further illustrated by the work of Wang et al. [36], who reviewed various markers that can indicate honey’s botanical and geographical origins, including phenolic compounds and organic acids. These markers can provide valuable information about the honey’s source and quality, which is essential for both consumers and producers. Additionally, advanced analytical techniques, such as mass spectrometry and chemometric modeling, are effective methods for classifying honey based on its geographical and botanical origins [37,38,39,40].

**Table 1:** Types of honey with their geographic origins and major uses.

Type of Honey	Primary Floral Source	Region Found	Characteristics	Major Uses/Forms of Usage	References
Manuka Honey	*Leptospermum scoparium*	New Zealand, Australia	Dark color, earthy flavor, high methylglyoxal content with potent antibacterial properties.	Medicinal uses include wound healing and immune support, and it is consumed raw.	[41,42]
Acacia Honey	*Robinia pseudoacacia* (Black Locust)	Europe, North America, Asia	Light color, mild flavor, and slow crystallization due to high fructose content.	Sweetener for tea, baking, and general cooking.	[43]
Clover Honey	*Trifolium* spp. (Clover)	United States, Canada, Europe	Light amber color, sweet floral flavor, commonly used in cooking and baking.	Spread on bread, salad dressings, sauces, and marinades.	[44]
Eucalyptus Honey	Eucalyptus trees	Australia, South Africa, Mediterranean	Medium to dark amber color, strong woody flavor, rich in antioxidants and antimicrobial properties.	Cough remedies, soothing sore throats, and used in herbal teas.	[43,45]
Buckwheat Honey	*Fagopyrum* spp. (Buckwheat)	United States, Canada, Eastern Europe	Dark color, robust flavor, and high antioxidant content.	Antioxidant-rich dietary supplement, baking, and adding to porridges.	[46,47]
Orange Blossom Honey	*Citrus* spp. (Orange trees)	United States (Florida, California), Spain	Light amber color, citrusy aroma and flavor, delicate sweetness.	Glazing for meats, desserts, beverages, and salad dressings.	[48]
Heather Honey	*Calluna vulgaris*	Scotland, Ireland, Scandinavia	Amber to dark red color, thick consistency, aromatic with earthy and slightly bitter notes.	Medicinal uses, cheese pairings, and raw consumption.	[49]
Sidr Honey	*Ziziphus* spp. (Jujube trees)	Yemen, Saudi Arabia, India	Dark amber color, rich flavor, highly prized for medicinal properties.	It is consumed for health benefits and energy boosting and mixed with herbal remedies.	[50,51]
Wildflower Honey	Mixed wildflower species	Worldwide	Varies in color and flavor based on regional floral sources and seasonality.	General sweetener, beverages, and versatile use in desserts and spreads.	[52,53]
Lavender Honey	*Lavandula* spp. (Lavender)	France, Spain, Italy	Light amber color, fragrant floral flavor, mild and delicate taste.	Aromatherapy, desserts, herbal teas, and natural skincare products.	[54]
Tupelo Honey	Nyssa ogeche (Tupelo trees)	Southeastern United States (Florida, Georgia)	Light golden color, buttery flavor, and high fructose content prevent crystallization.	Sweetener for beverages, gourmet cooking, and served with cheeses.	[55]
Neem Honey	*Azadirachta indica* (Neem tree)	India, Southeast Asia, Africa	Dark amber color, bitter taste, known for antibacterial and antifungal properties.	Medicinal uses, skincare, and health tonics.	[56,57]
Sage Honey	*Salvia* spp. (Sage plants)	California, Mediterranean regions	Light color, mild taste, slow to crystallize.	Tea sweetener, baking, and enhancing savory dishes.	[58,59]
Coffee Honey	*Coffea* spp. (Coffee plants)	Latin America, Africa, Southeast Asia	Dark amber color, bold and rich flavor with unique undertones from coffee blossoms.	Sweetening coffee beverages, baking, and used in desserts.	[60]

The use of honey in various applications, particularly in medicine and food science, has been well-documented in the literature [61,62,63]. Honey’s unique properties, including its antimicrobial effects, nutritional value, and versatility as an ingredient, make it a valuable resource in both traditional and modern contexts. In wound healing, honey has been extensively studied for its efficacy. Research by Tan et al. [62] highlights the role of Gelam honey in accelerating wound healing, attributing this effect to its production of hydrogen peroxide and its nutritional, hygroscopic, antioxidant, and antibacterial properties. Similarly, Khoo et al. [63] demonstrated that Tualang honey significantly enhances wound contraction and promotes granulation tissue formation in burn wounds, showcasing its effectiveness compared to conventional treatments. The antibacterial properties of honey are crucial in preventing infections, as noted by Lü et al. [64], who emphasized that honey can be as effective as conventional treatments in managing wounds, particularly in challenging cases such as diabetic ulcers and severe burns. Moreover, the case report by Saputri et al. [65] illustrated the successful application of local honey in treating a submandibular abscess, further supporting honey’s role in wound care. Beyond wound healing, honey’s nutritional contributions are noteworthy. Álvarez-Suarez et al. [66] provided a comprehensive review of honey’s benefits, emphasizing its historical use as both a food and a medicine, with applications ranging from treating burns to providing essential nutrients. Honey is rich in carbohydrates, vitamins, and minerals, making it a beneficial addition to various food products. Leite et al. [67] found that adding honey to beverages enhances flavor and improves the phenolic compound profile, contributing to the overall health benefits of the drink.

Furthermore, honey’s role as a natural sweetener and flavor enhancer in the beverage industry has been recognized, with its high fructose and glucose content providing sweetness and antioxidant properties [68,69,70]. The fermentation of honey into alcoholic beverages, such as mead, is another significant application. Honey’s composition makes it an excellent substrate for fermentation, as Ziuzia et al. [69] noted, when they reported high fermentation yields by using honey in mead production. The production of honey spirits and other fermented beverages is gaining popularity, as highlighted by Anjos et al. [70], who discussed the potential of honey to enhance the value of alcoholic drinks. Additionally, the unique flavor profiles and health benefits associated with honey-based beverages are being explored, with studies indicating that honey can contribute positively to the sensory characteristics of these products [71,72,73].

## 3. Chemical Composition of Honey

Honey is a complex natural substance produced by bees from the nectar of flowers or honeydew, which is the excretion of sap-sucking insects. Its composition is primarily characterized by a high concentration of sugars, predominantly fructose and glucose, which account for approximately 95% of honey’s dry weight [74]. The remaining constituents include water, enzymes, amino acids, vitamins, minerals, and various phytochemicals such as flavonoids and phenolic acids [75]. Essential oils derived from plants like tea tree, eucalyptus, and oregano are also widely studied for their ability to combat bacteria, fungi, and viruses. These natural agents disrupt microbial cell walls, inhibit biofilm formation, or interfere with microbial DNA replication. The specific proportions of these components can vary significantly depending on the floral source, geographical location, and environmental conditions under which the honey is produced [76,77]. Table 2 provides an overview of the diverse biochemical compounds present in honey, highlighting their sources and associated health benefits. Honey’s complex composition, derived from floral nectars, pollen, and bee enzymatic processes, makes it a uniquely beneficial natural product [35]. From essential sugars that provide energy to antioxidants and antimicrobial agents that support health and immunity, each class of compounds contributes to honey’s multifaceted role in human health. These compounds work synergistically to enhance honey’s nutritional and therapeutic value [78,79].

The sugar composition of honey is crucial for its properties and uses. Fructose is the most abundant sugar, followed by glucose, contributing to honey’s sweetness and energy content [80]. Other sugars, such as sucrose and turanose, are in smaller quantities and can influence the honey’s flavor and texture [81]. The presence of these sugars provides energy and plays a role in the stability of honey against fermentation, as higher sugar concentrations inhibit microbial growth [74]. Additionally, the moisture content, typically around 17–20%, is a critical factor in determining honey’s quality and shelf life [82]. Enzymes are another vital component of honey, with glucose oxidase being one of the most significant. This enzyme catalyzes the conversion of glucose into gluconic acid and hydrogen peroxide, contributing to honey’s antimicrobial properties [15,83]. Hydrogen peroxide production is significant, as it acts as a natural preservative and enhances honey’s ability to inhibit the growth of various pathogens, including bacteria and fungi [15]. Moreover, the low pH of honey, usually between 3.2 and 4.5, further contributes to its antimicrobial activity by creating an unfavorable environment for microbial proliferation [15,83].

**Figure 1:**
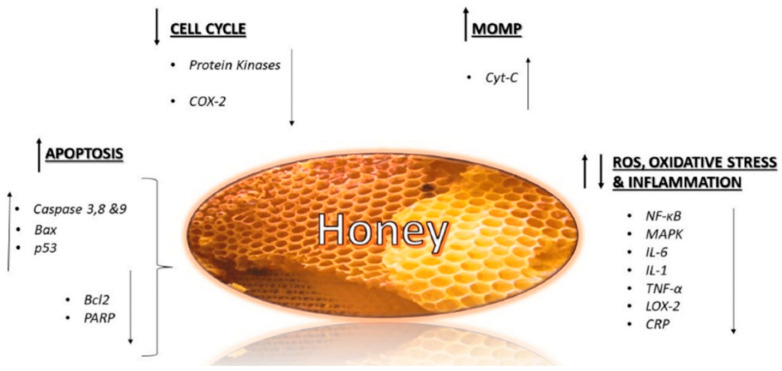
Phenolics in honey and their associated health benefits including their main mechanism of action and targets in the human cell. Source: Kunat-Budzyńska et al. [90].

Phytochemicals, including flavonoids and phenolic acids, are also present in honey and are responsible for many health benefits. These compounds exhibit antioxidant properties, which can help protect cells from oxidative stress and reduce the risk of chronic diseases [75,84]. Table 3 provides an overview of the antioxidants found in honey, highlighting their examples, sources, and associated health benefits. They work to neutralize free radicals, protect against chronic diseases, and promote cellular health, including phenolics (Figure 1). The diverse range of antioxidants in honey underscores its significance as a natural, multifunctional health-promoting agent. The concentration of these phytochemicals varies with the floral source of the honey, such as those derived from manuka or acacia, which are known for their higher antioxidant capacities compared to polyfloral varieties [36]. This variation underscores the importance of botanical origin in determining honey’s chemical profile and potential health benefits [85].

The floral source of honey influences its chemical composition and sensory attributes, including flavor, aroma, and color. Different flowers impart unique volatile compounds to honey, which can be used to determine its botanical origin [86]. For example, honey from citrus flowers may have distinct citrus notes, while those from wildflowers may exhibit more complex flavor profiles [86,87]. The volatile compounds in honey are also linked to its antimicrobial properties, as some possess inherent antibacterial activity [88].

Environmental factors, such as climate, soil composition, and local flora, play a significant role in shaping the chemical composition of honey. Honey produced in arid regions may have different mineral content and flavor profiles compared to honey from more temperate climates [76,77]. Additionally, the time of year, when the honey is harvested, can affect its composition, as seasonal variations in floral availability can lead to differences in nectar sources and, consequently, in the honey’s chemical makeup [76,77,89]. Honeydew honey, derived from the sugary excretions of aphids and other sap-sucking insects, presents a chemical profile different from floral honey. It typically contains higher levels of minerals and is darker, reflecting its unique botanical origin [90]. Specific compounds in honeydew honey can also influence its flavor and nutritional properties, making it distinct from blossom honey [90]. The quality of honey is often assessed through various physicochemical parameters, including moisture content, electrical conductivity, and specific enzymes and sugars [82]. The International Honey Commission has established guidelines for these quality criteria, which help ensure that honey meets specific standards for purity and safety [82]. Furthermore, detecting adulteration, such as adding sugars or other substances, is critical for maintaining honey’s integrity and consumer trust [74,91].

In addition to its nutritional and culinary uses, honey has been recognized for its therapeutic properties, particularly in traditional medicine and apitherapy. The antimicrobial and antioxidant activities of honey make it a valuable natural remedy for various ailments, including wounds and infections [15,84]. Research has shown that certain types of honey, such as Manuka honey, possess enhanced healing properties due to their unique chemical composition and higher concentrations of bioactive compounds [84,92]. The diverse applications of honey in food, medicine, and cosmetics highlight its significance as a natural product. Its rich chemical composition contributes to its flavor and sweetness and underpins its health benefits and therapeutic potential [75,84]. As consumers become more health conscious, the demand for high-quality, natural honey rises, prompting further research into its composition and properties [75,84].

**Table 2:** Major biochemical compounds in honey and their associated health benefits.

Class of Compounds	Examples	Sources in Honey	Health Benefits	References
Sugars	Glucose, fructose, and sucrose	Nectar of flowers	Provides energy, supports metabolic processes, and regulates blood glucose levels.	[43]
Water	~17% by weight	Natural hygroscopic properties	Maintains honey’s viscosity and influences shelf life.	[93]
Phenolic Compounds	Flavonoids (quercetin, kaempferol), and phenolic acids (gallic acid, and caffeic acid)	Derived from floral sources and plant resins	Acts as a powerful antioxidant, reducing oxidative stress, is anti-inflammatory, and supports cardiovascular health.	[94]
Proteins and Enzymes	Glucose oxidase, diastase, invertase, catalase	Bee salivary secretions and nectar	Antimicrobial properties, digestion enhancement, and immune system support.	[95]
Amino Acids	Proline, lysine, and glutamic acid	Proteinaceous pollen grains	Supports protein synthesis, cellular repair, and metabolic pathways.	[96]
Vitamins	Vitamin C, B-complex vitamins (B2, B6, and Niacin)	Nectar and pollen	Enhances immune function, promotes healthy skin, and supports energy metabolism.	[97]
Minerals	Potassium, calcium, magnesium, iron, zinc	Absorbed from soil via plant sources	Maintains electrolyte balance and supports bone density, oxygen transport, and enzymatic reactions.	[62]
Antioxidants	Ascorbic acid (vitamin C) and carotenoids	Natural components of nectar and pollen	Neutralizes free radicals, reduces cellular damage, and lowers inflammation levels.	[97]
Organic Acids	Gluconic acid, acetic acid, and citric acid	Enzyme activity in nectar	Antimicrobial effects aid digestion and contribute to honey’s acidic pH.	[35,98]
Lipids and Volatiles	Terpenes, and aldehydes	Flower resins and nectars	Provides unique flavor and aroma, supports sensory qualities, and offers antimicrobial properties.	[88]
Microbial Inhibitors	Hydrogen peroxide and methylglyoxal (in Manuka honey)	Derived from enzyme reactions	Antibacterial properties, especially effective against resistant bacterial strains.	[16,99]
Pollen Particles	Proteins, lipids, and other phytochemicals	From plants through bee activity	Source of proteins, immune-boosting compounds, and potential anti-allergic properties.	[25,100]
Trace Elements	Selenium, chromium, and copper	Trace quantities from environmental sources	Supports enzymatic functions, antioxidant defense, and metabolic regulation.	[101]

**Table 3:** Antioxidants in honey and their sources.

Antioxidant	Examples	Source in Honey	Health Benefits	References
Flavonoids	Quercetin, kaempferol, and Aapigenin	Nectar and pollen	Neutralizing free radicals reduce oxidative stress and the risk of chronic diseases such as heart disease and cancer.	[94,102]
Phenolic Acids	Gallic acid, caffeic acid, and ferulic acid	Plant-derived phenolic compounds	Anti-inflammatory properties promote cellular health and protect against neurodegenerative diseases.	[79,94]
Ascorbic Acid (Vitamin C)	Ascorbic acid	Nectar and enzymatic reactions	Boosts the immune system, reduces inflammation, and supports skin health by promoting collagen synthesis.	[78,97]
Carotenoids	Beta-carotene and lycopene	Derived from floral nectar	Support eye health, enhance skin protection, and serve as precursors to Vitamin A.	[103,104]
Enzymatic Antioxidants	Catalase, and glucose oxidase	Bee-secreted enzymes	Generates hydrogen peroxide, which has antimicrobial properties and supports oxidative balance.	[16]
Tocopherols	Alpha-tocopherol (vitamin E)	Natural components of plant nectars	Protects cell membranes from oxidative damage and supports cardiovascular health.	[105]
Organic Acids	Gluconic acid, and citric acid	Byproducts of enzymatic activity	Maintain honey’s acidic pH, which has antimicrobial properties and contributes to overall oxidative balance.	[102,106]
Methylglyoxal	Found in Manuka honey	Enzymatic modification of nectar	Potent antibacterial properties and support in managing resistant bacterial strains.	[42]
Trace Elements	Selenium and zinc	Soil-derived through plants	Contribute to antioxidant defense mechanisms and enzymatic functions.	[107]

## 4. Mechanisms of Honey’s Antimicrobial Action

**Figure 2:**
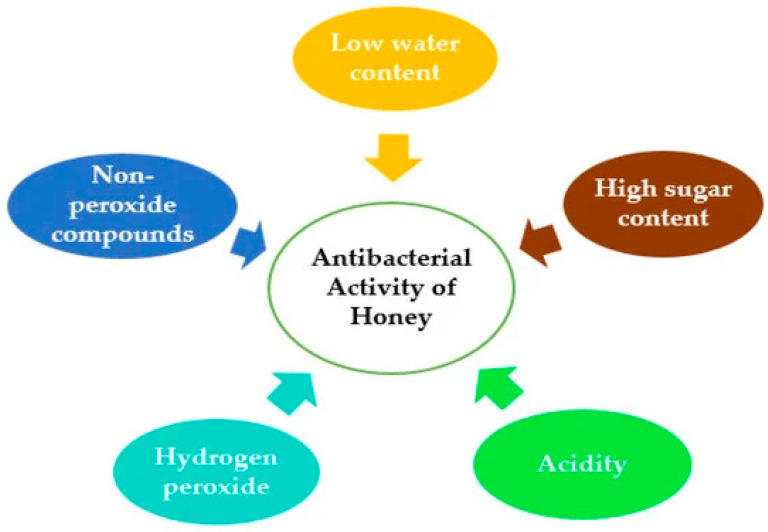
Parameters that contribute to the antimicrobial potential of honey. Source: Hossain et al. [118].

Honey has been recognized for its medicinal properties for centuries, particularly its antimicrobial effects. Honey’s remarkable antimicrobial properties are derived from a combination of physical, chemical, and biochemical mechanisms that synergize to inhibit microbial growth and survival (Table 4; Figure 2). Its high sugar concentration, low water activity, and acidic pH create an environment that is hostile to most pathogens. Honey’s enzymatic activity also produces hydrogen peroxide and other bioactive compounds, such as methylglyoxal and polyphenols, which exhibit potent antimicrobial effects. These mechanisms target microbial cells directly and disrupt processes like biofilm formation and protein synthesis. The table below provides a comprehensive overview of the key mechanisms of honey’s antimicrobial action, the components involved, and their impact on microbial activity, showcasing its potential as a natural and effective antimicrobial agent in medical and food applications. One of the primary mechanisms is the production of hydrogen peroxide (H_2_O_2_), which is generated by the enzyme glucose oxidase in honey. This enzyme catalyzes the breakdown of glucose into hydrogen peroxide in the presence of water, creating a potent antimicrobial environment. The H_2_O_2_ produced acts as a reactive oxygen species, damaging bacterial cell structures, proteins, and DNA, ultimately leading to cell death [15,108]. Notably, the concentration of hydrogen peroxide in honey is relatively low, which allows it to be safe for topical application on wounds while effectively inhibiting bacterial growth [16]. The sustained release of hydrogen peroxide over time further enhances honey’s ability to prevent infections in wounds and cuts, making it a valuable therapeutic agent [15,108].

In addition to hydrogen peroxide, the high sugar concentration in honey, particularly in fructose and glucose, plays a crucial role in its antimicrobial properties. The osmotic effect of honey creates a hypertonic environment that draws water out of microbial cells, leading to dehydration and eventual cell death [16,109]. This high osmolarity inhibits microbial growth and disrupts the cellular functions of bacteria, fungi, and other pathogens such as viruses like herpes simplex virus and protozoa like *Leishmania* species. In wound care, honey’s ability to reduce moisture levels creates an environment that is less conducive to the growth of many pathogens, thereby aiding in the prevention of infections [15,16]. Combining these mechanisms highlights honey’s multifaceted approach to combating microbial threats, making it a unique and effective natural remedy. Antioxidants present in honey also contribute significantly to its antimicrobial effects. Honey contains various antioxidants, including flavonoids, phenolic acids, and ascorbic acid, which are known to neutralize harmful free radicals [36,110]. These antioxidants can inhibit the growth of bacteria and fungi by damaging microbial cell membranes and interfering with their metabolic processes. Moreover, studies have indicated that the antioxidant properties of honey may enhance its antimicrobial effects by boosting the immune response in the body, further supporting its role in wound healing [36,110]. By reducing oxidative stress and promoting tissue repair, honey’s antioxidants contribute to its antimicrobial action and facilitate wound healing [36,111,112].

The ability of honey to disrupt biofilms is another critical aspect of its antimicrobial action. A biofilm is a structured community of microorganisms, such as bacteria or fungi, that adhere to a surface and are embedded within a self-produced matrix of extracellular polymeric substances. This matrix provides protection against environmental stressors, antibiotics, and the host immune system, making biofilms highly resistant to treatments and contributing to persistent infections. They are clusters of bacteria embedded in a protective matrix, making them more resistant to antibiotics and other antimicrobial treatments. Honey has been shown to effectively break down biofilms, making bacteria more susceptible to its direct antimicrobial action and conventional antibiotics [113,114]. This is particularly important in chronic infections, where biofilms protect bacteria from immune system clearance and treatment [113,114]. The inhibition of biofilm formation by honey is attributed to its low molecular weight components and specific proteins, such as major royal jelly protein 1 (MRJP1), which have been identified as key players in this process [113,115]. This ability to combat biofilms underscores honey’s potential as a therapeutic agent against antibiotic-resistant strains, such as Methicillin-resistant *S. aureus* (MRSA) [16,114]. Methylglyoxal (MGO), a compound found in significant concentrations in Manuka honey, is another crucial contributor to honey’s antimicrobial effects. MGO is produced through the conversion of dihydroxyacetone (DHA), a compound found in the nectar of the Manuka plant (*Leptospermum scoparium*), into MGO [15,114]. Research has demonstrated that MGO exhibits broad-spectrum antimicrobial activity against Gram-positive and Gram-negative bacteria, certain fungi, and viruses [15,114]. MGO is one of the distinguishing factors that sets Manuka honey apart from other types of honey, linking it to its higher antimicrobial potency and therapeutic efficacy [15,110]. This unique characteristic of Manuka honey has garnered significant attention in clinical and research settings, further validating its use as a natural antimicrobial agent.

The antimicrobial properties of honey are not limited to its chemical composition; the botanical origin of honey also plays a crucial role in determining its efficacy. Different floral sources yield honey with varying antimicrobial activities, influenced by the specific phytochemicals present in the nectar [116]. For instance, honey derived from certain plants may contain higher concentrations of antioxidants or antimicrobial compounds, leading to enhanced efficacy against pathogens [117]. This variability underscores the importance of understanding the botanical origin of honey when evaluating its potential therapeutic applications. Studies have shown that honey from diverse geographic and botanical sources can exhibit significant differences in their ability to inhibit the growth of bacteria and fungi, further emphasizing the need for careful selection of honey types for medicinal use [116,117]. Moreover, the antimicrobial efficacy of honey has been extensively studied in the context of wound healing. Various studies have reported that honey can effectively reduce the incidence of infections in chronic wounds and promote faster healing compared to conventional treatments [65,118]. The unique combination of antimicrobial properties and its ability to maintain a moist wound environment makes honey an attractive alternative to traditional antibiotics, particularly in the face of rising antibiotic resistance [65,118]. The application of honey in wound care has been supported by numerous clinical trials, demonstrating its effectiveness in treating a wide range of wound types, including burns, surgical wounds, and diabetic ulcers [65,118].

The Minimum Inhibitory Concentration (MIC) of honey—the lowest concentration required to inhibit visible microbial growth—varies depending on factors such as the type of honey, its floral source, and the specific microorganism being tested. Studies have reported MIC values ranging from as low as 1% to over 50% (weight/volume) [119,120,121,122,123,124]. For instance, potent honey like Manuka, dark buckwheat, heather, or molasses have demonstrated MIC values between 1% and 12.5% (*w*/*v*), while lighter-colored honey such as clover or acacia exhibits MIC values ranging from 25% to 50% (*w*/*v*) [123]. Additionally, research on Sidr honey and Manuka honey against *E. coli* found MICs of 50% and 30%, respectively [124]. These variations underscore the importance of considering specific honey types and target pathogens when evaluating honey’s antimicrobial efficacy.

In addition to its antimicrobial properties, honey has been shown to modulate the inflammatory response, further contributing to its wound-healing capabilities. The anti-inflammatory effects of honey can help reduce swelling and pain associated with wounds, promoting a more conducive environment for healing [119]. This dual action of lowering microbial load while modulating inflammation highlights honey’s multifaceted role in wound management. Furthermore, honey’s ability to stimulate tissue regeneration and angiogenesis has been documented, suggesting that it prevents infection and promotes healing [119]. The safety profile of honey, particularly in its topical application, is another significant advantage. Unlike many conventional antibiotics, honey has a low propensity for inducing resistance in bacteria, making it a promising option for treating infections caused by antibiotic-resistant strains [16,114]. This characteristic is fundamental in the context of rising antibiotic resistance, where the need for alternative therapeutic strategies is becoming increasingly urgent [16,114]. The historical use of honey in traditional medicine, coupled with modern scientific validation of its antimicrobial properties, positions honey as a valuable resource in the fight against infections, particularly in wound care settings [16,114].

**Table 4:** Mechanisms by which honey inhibits microbial growth and survival.

Mechanism	Characteristics	Key Components Involved	Impact on Microbial Activity	References
Low Water Activity	Honey’s high sugar concentration creates an osmotic effect, drawing water out of microbial cells.	Glucose, fructose	Dehydrates and inhibits microbial growth.	[106]
Acidic pH	Honey’s natural acidic pH (3.2 to 4.5) creates an inhospitable environment for most pathogens.	Organic acids (e.g., Gluconic acid)	Prevents the growth and survival of many bacteria and fungi.	[78]
Hydrogen Peroxide Production	Enzymatic activity generates hydrogen peroxide, which has potent antimicrobial effects.	Glucose oxidase	Damages microbial cells, disrupts DNA, and impairs replication.	[121]
Methylglyoxal	This compound disrupts microbial metabolism and integrity when found in specific honey (e.g., Manuka).	Methylglyoxal	Targets bacterial cell walls and protein synthesis.	[42]
High Sugar Concentration	The dense sugar content disrupts microbial cell function by interfering with nutrient and water intake.	Glucose, fructose	Inhibits energy production and growth in microbes.	[122]
Presence of Polyphenols	Flavonoids and phenolic acids possess antioxidant and antimicrobial properties.	Quercetin, gallic acid, caffeic acid	Neutralizes free radicals and inhibits microbial enzymes.	[79]
Aromatic Compounds	Volatile compounds from floral sources have antimicrobial effects.	Terpenes, aldehydes, ketones	Inhibits growth and reduces microbial activity.	[88]
Low Protein Content	Limited nitrogen availability in honey reduces the microbial capacity for protein synthesis.	Naturally low protein levels	Starves microbes of necessary building blocks.	[123]
Biofilm Disruption	Honey interferes with the formation and stability of microbial biofilms, preventing colonization.	Multiple synergistic components	Reduces microbial persistence and resistance.	[114]
Direct Toxic Effects	Certain compounds in honey exhibit direct toxic effects on specific pathogens.	Phenols, organic acids, hydrogen peroxide	Kills microbes and prevents proliferation.	[124]

## 5. Types of Honey with Antimicrobial Properties: Case Study Analysis

Honey, a natural sweetener produced by bees, is cherished for its flavor and diverse health benefits, particularly its antimicrobial properties. Among the various types of honey, some have gained prominence due to their unique compositions and potent antimicrobial activities. Manuka honey, derived from the nectar of the *L. scoparium* plant, primarily found in New Zealand and parts of Australia, is renowned for its exceptional antimicrobial properties. The unique compounds present in Manuka honey, particularly MGO, contribute significantly to its antibacterial efficacy. Studies have shown that Manuka honey can inhibit Gram-positive and Gram-negative bacteria growth, including antibiotic-resistant strains such as methicillin-resistant *S. aureus* (MRSA) [125,126]. The UMF rating system is often employed to quantify the antimicrobial strength of Manuka honey, with higher UMF values indicating greater concentrations of beneficial compounds [125]. This honey’s ability to combat infections has led to its widespread recognition and use in medical applications, especially in wound care [125,126].

In addition to Manuka honey, thyme honey also exhibits significant antimicrobial potential. Derived from the nectar of thyme plants, this honey is rich in antioxidants and has demonstrated effectiveness against various pathogens, including *S. aureus* and *E. coli* [83,127]. Flavonoids and phenolic acids in thyme honey enhance its antibacterial action, making it a valuable natural remedy for infections [127]. Research indicates that thyme honey’s antimicrobial properties are comparable to those of Manuka honey, particularly in its ability to inhibit the growth of resistant bacterial strains [127]. Moreover, thyme honey’s antioxidant properties further contribute to its health benefits, as they help combat oxidative stress and inflammation [128].

Eucalyptus honey, sourced from the flowers of eucalyptus trees, is another type of honey recognized for its potent antimicrobial and anti-inflammatory properties. This honey has shown effectiveness against respiratory pathogens, making it particularly beneficial for treating coughs, colds, and upper respiratory infections [83]. Eucalyptus honey exhibits antibacterial activity against various strains, including Streptococcus and *Staphylococcus* species [83,106]. While its antimicrobial strength may not match Manuka or thyme honey, eucalyptus honey remains popular for its soothing properties and ability to support respiratory health [83].

Clover honey, one of the most common varieties, is derived from the nectar of clover flowers. Although its antimicrobial properties are less potent than those of Manuka or thyme honey, clover honey still demonstrates moderate antibacterial and antifungal effects [83,106]. Research supports its use in wound care, highlighting its ability to accelerate healing and reduce the risk of infection [83]. The mild antimicrobial action of clover honey makes it suitable for general wellness applications, providing a gentle yet effective option for those seeking natural remedies [83]. When comparing the antimicrobial strengths of these honey types, it is evident that Manuka honey stands out as the most potent due to its high concentration of MGO and other bioactive compounds [125]. Thyme honey follows closely, particularly in its effectiveness against a broad spectrum of bacteria, including Gram-positive and Gram-negative strains [83,127]. Eucalyptus honey, while effective against respiratory pathogens, may be less potent against gastrointestinal bacteria than Manuka or thyme honey [83]. Although widely used, clover honey has a relatively milder antimicrobial action and is often utilized for general wellness rather than as a potent antibacterial agent [83].

The role of methylglyoxal (MGO) in Manuka honey is crucial to understanding its antimicrobial properties. MGO is a naturally occurring compound in high concentrations in Manuka honey, responsible for much of its antibacterial activity [125]. The antibacterial effect of MGO operates by disrupting bacterial cell processes, including protein synthesis and cell wall formation, ultimately leading to bacterial death [125]. The concentration of MGO in Manuka honey correlates directly with its antimicrobial strength, with higher levels providing more decisive antibacterial action [125]. Additionally, MGO is recognized for its anti-inflammatory and antioxidant properties, further enhancing the therapeutic benefits of Manuka honey, particularly in wound healing and skin care applications [125]. Honey’s anti-inflammatory properties are primarily mediated through its ability to modulate immune responses and reduce oxidative stress. Key bioactive compounds, including flavonoids, phenolic acids, and MGO, play a role in downregulating pro-inflammatory cytokines, such as tumor necrosis factor-alpha (TNF-α), interleukin-6 (IL-6), and interleukin-1 beta (IL-1β). Honey also inhibits the nuclear factor-kappa B (NF-κB) signaling pathway, a crucial regulator of inflammation, thereby reducing the expression of inflammatory mediators [119]. Additionally, its high antioxidant content helps neutralize reactive oxygen species, which contribute to tissue damage and prolonged inflammation. These combined effects result in decreased swelling, pain relief, and enhanced wound healing. The antimicrobial mechanisms of honey are multifaceted and involve several factors beyond MGO. Honey’s high osmolarity and low pH create an inhospitable environment for bacteria, while hydrogen peroxide contributes to its antibacterial effects [129]. Non-peroxide components, such as flavonoids and phenolic compounds, also significantly affect honey’s antimicrobial activity [129]. For instance, thyme honey’s effectiveness against methicillin-resistant *S. aureus* has been attributed to its unique phytochemical composition, which enhances its antibacterial properties [83,127]. This complexity underscores the importance of considering the specific composition of each honey type when evaluating its antimicrobial potential.

Different types of honey exhibit varying antimicrobial properties, influenced by their botanical origin, chemical composition, and geographical factors. Manuka honey, known for its high MGO content, has demonstrated strong antibacterial effects with MIC values ranging from 2.5% to 10% (*v*/*v*) against *S. aureus*, *E. coli*, and *P. aeruginosa* [126]. Thyme honey, rich in flavonoids and phenolic acids, has shown MIC values of 3% to 12% (*v*/*v*) against *Salmonella enterica* and *Listeria monocytogenes* [128]. Sidr honey (*Ziziphus* sp.) is known to exhibit antibacterial activity against *Helicobacter pylori* with high MIC values. Chestnut honey, due to its high polyphenol content, also has MIC values of 4% to 10% against *Enterococcus faecalis* and *Streptococcus pneumoniae* [108]. Although generally less potent, clover honey inhibits *Bacillus cereus* and *E. coli* at concentrations of 8% to 20% [127]. These findings highlight the diverse antimicrobial potential of different honey types, reinforcing their role as natural antibacterial agents.

Research has demonstrated that honey can effectively combat antibiotic-resistant pathogens, making it a valuable alternative or adjunct to conventional antibiotics [126,130]. For example, studies have shown that Manuka honey can disrupt biofilms formed by resistant bacteria, enhancing the efficacy of antibiotics when combined [126]. This synergistic effect suggests that honey may help mitigate the growing issue of antibiotic resistance by providing a natural means of infection control [126]. Furthermore, the diverse antimicrobial properties of various honey types highlight the potential for developing new therapeutic strategies incorporating honey as a natural antimicrobial agent [126,130].

## 6. Clinical and Laboratory Evidence of the Antimicrobial Role of Honey

Honey has been recognized for its medicinal properties for centuries, and recent clinical and laboratory studies have provided substantial evidence supporting its effectiveness against various pathogens. The antimicrobial properties of honey are attributed to its unique composition, which includes high sugar content, low pH, and the presence of hydrogen peroxide and other phytochemicals. These factors contribute to honey’s ability to inhibit the growth of a wide range of microorganisms, including bacteria, fungi, and viruses. Numerous studies have documented honey’s efficacy against antibiotic-resistant strains, making it a valuable alternative in the fight against infections that are increasingly difficult to treat with conventional antibiotics [16,131,132]. The effectiveness of honey as a topical treatment for wounds has been particularly well-documented. Clinical trials have shown that honey can significantly enhance the healing process for chronic wounds, such as diabetic ulcers and burn injuries [133,134]. A multicenter observational study indicated that honey-treated wounds exhibited a notable reduction in size and improved healing rates compared to those treated with standard antiseptics [135,136]. The mechanisms through which honey promotes wound healing include its antibacterial properties, ability to maintain a moist wound environment, and stimulation of tissue regeneration processes such as angiogenesis and epithelialization [137].

Honey’s activity against antibiotic-resistant bacteria is an area of growing interest, especially considering the global rise in antimicrobial resistance. Research has demonstrated that honey can effectively inhibit the growth of multi-drug resistant pathogens, including methicillin-resistant *S. aureus* and various strains of *Escherichia coli* [16]. The presence of methylglyoxal, particularly in Manuka honey, has been identified as a key factor contributing to its potent antibacterial activity [16]. This characteristic makes honey a promising adjunctive therapy for infections caused by resistant strains, potentially reducing the reliance on traditional antibiotics. In addition to its antibacterial properties, honey is effective against fungal infections, particularly those caused by *Candida* species. Studies have indicated that honey can inhibit the growth of *Candida albicans*, a common pathogen responsible for oral thrush and systemic infections in immunocompromised individuals [16]. Furthermore, honey’s ability to disrupt fungal biofilms, often resistant to conventional antifungal treatments, highlights its potential as a therapeutic agent in managing fungal infections [16]. Laboratory studies have played a crucial role in elucidating honey’s antimicrobial properties. In vitro experiments have consistently demonstrated honey’s ability to inhibit a wide array of pathogens, with varying degrees of effectiveness depending on the type of honey and its concentration [138]. For example, research has shown that specific floral honey sources exhibit more potent antibacterial activity than others, with Manuka honey often cited as the most powerful [16]. However, the variability in honey’s composition due to factors such as floral source, geographical location, and processing methods can complicate the interpretation of results across studies [16,139].

Clinical trials have further validated honey’s effectiveness in treating various infections, particularly wound care. A systematic review of clinical trials indicated that honey-treated wounds exhibited faster healing times and lower infection rates than conventional treatments [136]. Despite these promising findings, some clinical trials have faced limitations, including small sample sizes and inconsistent honey quality, which can impact the generalizability of results [135,136]. Specific pathogens have been the focus of numerous studies investigating honey’s antimicrobial effects. For instance, honey has demonstrated significant activity against *S. aureus*, including MRSA and *P. aeruginosa*, common culprits in wound infections [16]. Additionally, honey has shown inhibitory effects against *H. pylori*, the bacterium responsible for peptic ulcers, suggesting its potential role in gastrointestinal health [135].

While less established than its antibacterial and antifungal effects, honey’s antiviral properties have also garnered attention. Preliminary studies suggest that honey may possess antiviral activity against respiratory viruses, including those responsible for the common cold and influenza [16]. The proposed mechanisms for these effects include honey’s antioxidant properties and ability to modulate the immune response, although further research is required to substantiate these claims [16]. Honey’s ability to disrupt biofilms is particularly noteworthy, as biofilm formation is a significant factor in the persistence of chronic infections and antibiotic resistance. Studies have indicated that honey can effectively disrupt biofilms produced by bacteria such as *P. aeruginosa* and *S. aureus*, making it a potential adjunctive therapy for chronic infections [16,139]. These characteristics underscore honey’s multifaceted role in infection management, as it inhibits microbial growth and addresses the challenges posed by biofilm-associated infections.

Despite the promising evidence supporting honey’s antimicrobial properties, several limitations persist in the current body of research. Inconsistent results across studies can be attributed to variations in honey quality, including contamination and processing methods, which can influence its effectiveness [16]. Additionally, the lack of standardization in honey types and concentrations used in studies complicates the comparison of results and the establishment of definitive clinical guidelines for honey’s use [16]. The need for more extensive, well-controlled clinical trials is evident to further elucidate honey’s therapeutic potential and establish clear guidelines for its application in clinical practice. While existing studies provide a foundation for understanding honey’s effectiveness against pathogens, more robust research is necessary to address the limitations identified in previous trials and to explore the full spectrum of honey’s medicinal properties [16,135].

## 7. Applications in Healthcare and Public Health for the Antimicrobial Effects of Honey

Honey has been utilized for centuries in various cultures for its therapeutic properties, and its applications in healthcare and public health are increasingly being recognized in modern medicine. The multifaceted benefits of honey stem from its unique composition, which includes sugars, vitamins, minerals, and various bioactive compounds that contribute to its antimicrobial, anti-inflammatory, and antioxidant properties [140,141]. Some of the diverse applications of honey in healthcare primarily focus on its use in wound healing, gastrointestinal disorders, combating antibiotic-resistant bacteria, oral health, and the contrast between traditional and modern medical uses.

One of the most prominent applications of honey in healthcare is its role in wound healing and the prevention of infections. Honey has been shown to possess broad-spectrum antimicrobial activity, making it an effective topical treatment for various types of wounds, including surgical wounds, burns, and ulcers [142,143]. The mechanism behind honey’s effectiveness lies in its high osmolarity, which creates a hypertonic environment that inhibits bacterial growth and the production of hydrogen peroxide and other antimicrobial compounds [106,141]. Studies have demonstrated that honey can significantly reduce healing time and inflammation in infected wounds, often showing comparable results to conventional topical antibiotics [142,143]. Furthermore, honey’s ability to stimulate epithelial development and angiogenesis enhances wound healing, making it a valuable adjunct in modern wound care protocols [142,144].

In addition to its wound-healing properties, honey has been explored as a treatment for gastrointestinal disorders, such as ulcers and gastroenteritis. Research indicates that honey can be beneficial in managing symptoms associated with these conditions, particularly in children. Studies have found that adding honey to oral rehydration solutions improved treatment outcomes for gastroenteritis in infants and children, demonstrating its antibacterial properties against pathogens responsible for diarrhea [145,146,147]. Honey’s natural composition, including its antioxidant and anti-inflammatory properties, may also contribute to the healing of gastric mucosa and the reduction in ulcer symptoms [141,148]. These findings highlight honey’s potential as a safe and effective alternative or adjunct therapy in gastrointestinal health.

The rise in antibiotic-resistant bacteria has prompted researchers to investigate honey as a potential alternative treatment. Honey exhibits significant antibacterial activity against a variety of pathogens, including multidrug-resistant strains such as Methicillin-resistant *S. aureus* and *P. aeruginosa* [148,149]. Studies have shown that honey not only inhibits the growth of these resistant bacteria but also prevents the formation of biofilms, which are often associated with chronic infections [114,150]. The unique combination of sugars, phenolic compounds, and other bioactive components in honey contributes to its effectiveness against antibiotic-resistant bacteria, suggesting that it could be integrated into treatment regimens to enhance antimicrobial efficacy and reduce reliance on conventional antibiotics [106,151].

Honey’s role in oral health is another area of growing interest. Its antibacterial properties effectively manage oral cavity infections, gingivitis, and other periodontal diseases. Research has indicated that honey can inhibit the growth of oral pathogens, thereby reducing plaque formation and gingival bleeding [144,152]. Additionally, honey’s anti-inflammatory properties may help alleviate symptoms associated with oral mucositis, particularly in patients undergoing radiation therapy [144,153]. The use of honey in oral healthcare products, such as toothpaste and mouth rinses, is being explored as a natural alternative to conventional oral hygiene products, potentially improving overall oral health outcomes [152,154]. The contrast between traditional and modern medical uses of honey is noteworthy. Traditionally, honey has been employed in various cultures for its health benefits, often passed down through generations as a natural remedy for ailments ranging from coughs to wounds [155,156]. However, the advent of modern medicine and the development of synthetic antibiotics led to a decline in using honey as a therapeutic agent. Recent scientific investigations have reignited interest in honey’s medicinal properties, leading to a resurgence in its application in contemporary healthcare settings [63,157]. This shift reflects a growing recognition of the importance of integrating traditional remedies with modern medical practices, particularly in the context of rising antibiotic resistance and the need for alternative treatment options.

## 8. Honey in Public Health Strategies

Honey has garnered attention in public health strategies, including in traditional medicine, particularly in low-resource settings. Its integration into health programs is increasingly recognized for its potential infection prevention and treatment benefits. The antimicrobial properties of honey, attributed to its unique composition, make it a viable alternative to conventional antimicrobial treatments, especially in rural and underserved areas where access to healthcare may be limited. Studies have shown that honey can effectively reduce the risk of infections, such as endophthalmitis, during eye surgeries, highlighting its role as a prophylactic agent [158]. Furthermore, honey’s ability to inhibit bacterial growth, including strains resistant to antibiotics, positions it as a cost-effective solution for managing infections [159].

The antimicrobial efficacy of honey is well-documented, with various studies demonstrating its effectiveness against a range of pathogens. For instance, research has shown that honey can inhibit methicillin-resistant *S. aureus* and other resistant bacteria, making it a critical tool in the fight against antibiotic resistance [15]. The mechanisms behind honey’s antimicrobial action include its high sugar content, which creates an osmotic effect that inhibits microbial growth, and the presence of hydrogen peroxide and other bioactive compounds [76,160]. These properties contribute to honey’s effectiveness in wound healing and underscore its potential as a preventive measure in public health strategies. Using honey-based products can significantly enhance infection prevention efforts in rural and underserved areas, where healthcare resources are often scarce. Honey’s application in wound care, particularly in chronic wounds and surgical sites, has been shown to promote healing while minimizing the risk of infection [161]. For example, a study comparing honey to normal saline in managing postoperative infected wounds in pediatric patients demonstrated honey’s superior efficacy in promoting healing and preventing infection [162]. This is particularly relevant in low-resource settings where access to advanced medical treatments may be limited, making honey an attractive alternative.

The cost-effectiveness of honey as an antimicrobial treatment is another compelling reason for its integration into public health strategies. Conventional antimicrobial therapies can be expensive and often have side effects, including the risk of developing resistance [159]. In contrast, honey is relatively inexpensive and widely available, especially in regions with local beekeeping practices. Its use can reduce healthcare costs associated with treating infections, alleviating the financial burden on healthcare systems in low-resource settings [162]. Additionally, honey’s long shelf life and ease of storage further enhance its practicality as a public health intervention. Moreover, honey’s role in promoting overall health extends beyond its antimicrobial properties. It is rich in antioxidants, which can help combat oxidative stress and inflammation and improve health outcomes [163]. The antioxidative properties of honey have been linked to enhanced immune function, making it a valuable addition to public health initiatives aimed at improving community health, particularly in vulnerable populations [163]. By incorporating honey into dietary recommendations and health education programs, public health officials can promote its consumption as a natural means of enhancing health and preventing disease.

Combining honey into public health strategies also aligns with the growing interest in natural and alternative therapies. As consumers become more aware of the potential side effects of synthetic medications, there is an increasing demand for natural remedies that are perceived as safer and more effective [159]. Honey fits this trend perfectly with its historical use in traditional medicine and scientifically validated health benefits. Public health campaigns that educate communities about the benefits of honey can empower individuals to take charge of their health and make informed choices about their treatment options. In addition to its health benefits, honey production can contribute to local economies, particularly in rural areas where beekeeping is practiced. Public health initiatives can stimulate local economies and support sustainable agricultural practices by promoting honey as a health product. This dual benefit of improving health outcomes while fostering economic development makes honey an ideal candidate for inclusion in public health strategies [164]. Furthermore, promoting local honey can enhance community engagement and awareness of the importance of biodiversity and pollinator health, which are critical for sustainable food systems. Despite the promising potential of honey in public health, it is essential to consider the quality and source of honey used in health interventions. Variability in the antimicrobial properties of honey can arise from differences in floral sources, geographical location, and processing methods [109]. Therefore, public health programs should emphasize using high-quality, standardized honey products to ensure consistent therapeutic outcomes. Collaborations with local beekeepers and honey producers can help establish quality control measures and promote the use of locally sourced honey in health initiatives.

## 9. Challenges Associated with the Use of Honey as an Antimicrobial

Honey faces numerous challenges and considerations regarding its quality, safety, and efficacy. One of the primary challenges is the quality control and standardization of honey products. The variability in honey composition, influenced by factors such as floral source, geographic location, and production methods, complicates the establishment of uniform quality standards. The International Honey Commission has emphasized the need for harmonized analytical methods to certify honey quality and authenticity, particularly concerning its botanical origin and chemical composition [86]. The lack of standardized regulations can lead to inconsistencies in product quality, making it difficult for consumers to assess the reliability of the available honey products [165].

Another significant concern is the risk of contamination and adulteration in commercially available honey. Studies have shown that many honey samples exceed the acceptable limits for sucrose content, which can indicate adulteration with sugar syrups [166]. Furthermore, using high-fructose corn syrup (HFCS) in bee feeding has raised alarms regarding its potential negative impact on honey quality [167]. The adulteration of honey undermines consumer trust and poses health risks, as some contaminants may be unsafe for consumption. Rigorous testing and monitoring of honey products is paramount to ensure they meet safety standards and are free from harmful substances [168].

In addition to quality control issues, honey’s antimicrobial properties present limitations that warrant consideration. While honey is widely recognized for its antibacterial effects, particularly against specific pathogens, it is not universally effective against all microbial strains. Research has indicated that the antimicrobial activity of honey can vary significantly based on its floral source and processing methods [83]. Moreover, the mechanisms of action behind honey’s antimicrobial properties, such as the presence of hydrogen peroxide and methylglyoxal (MGO), may not be sufficient to combat antibiotic-resistant bacteria [16]. This limitation highlights the necessity for further research to explore the full spectrum of honey’s antimicrobial capabilities and its potential role in treating infections. Potential allergies and side effects of honey consumption also pose challenges, particularly for specific populations. Some individuals may experience allergic reactions to honey, especially those sensitive to pollen or bee products [16]. Additionally, the high sugar content in honey can be a concern for individuals with diabetes or those monitoring their sugar intake. As honey is often perceived as a natural sweetener, consumers must be aware of its caloric content and glycemic index, which can impact blood sugar levels [169]. Therefore, public health education regarding the appropriate consumption of honey is essential to mitigate these risks.

Quality control measures must also encompass honey’s physicochemical properties, which are critical indicators of its authenticity and quality. Parameters such as moisture content, diastase activity, and hydroxymethylfurfural presence are routinely analyzed to assess honey quality [170]. For instance, diastase activity is a key factor in determining honey’s freshness and storage conditions, with lower values indicating potential degradation [171]. Establishing clear quality standards based on these physicochemical characteristics ensures that honey products meet consumer expectations and regulatory requirements.

The diverse range of honey types available in the market further compounds the challenges of honey quality control. Different floral sources yield honey with distinct flavors, colors, and nutritional profiles, affecting consumer preferences and marketability [172]. The increasing demand for specialty honey, such as Manuka honey, has led to many products claiming unique health benefits, necessitating robust authentication methods to prevent misleading claims [173]. The quantification of specific compounds, such as leptosperin in Manuka honey, has emerged as a promising approach for verifying authenticity and quality [173,174].

Moreover, the global honey trade presents additional quality assurance and regulatory compliance challenges. The lack of a unified international standard for honey quality can create trade barriers, as different countries may impose varying regulations [165]. This inconsistency complicates the import and export of honey products, making it essential for producers to navigate complex regulatory landscapes to ensure compliance with local and international standards [168]. Establishing collaborative frameworks among countries could facilitate the development of standardized quality measures, enhancing consumer confidence in honey products. The environmental impact of honey production is another consideration that cannot be overlooked. Sustainable beekeeping practices are crucial for maintaining bee populations and ensuring the long-term viability of honey production. Using pesticides and monoculture farming can adversely affect bee health, leading to declines in honey production and biodiversity [165]. Promoting sustainable agricultural practices and supporting local beekeepers can help mitigate these environmental challenges while ensuring the quality and authenticity of honey products.

## 10. Future Directions and Research Needs to Enhance the Antimicrobial Value of Honey

The future directions and research needs concerning honey, particularly its antimicrobial potential, are increasingly becoming a focal point in clinical and laboratory settings. Recent studies have highlighted the broader therapeutic applications of honey, especially its efficacy against antibiotic-resistant bacteria such as methicillin-resistant *S. aureus* and Vancomycin-resistant *Enterococcus*. Brudzynski and Lannigan [106] and Masoura et al. [173] elucidated the mechanism behind honey’s bacteriostatic action, attributing it to hydroxyl radicals generated from hydrogen peroxide, a key antimicrobial component in honey. This finding underscores the necessity for further exploration into the specific pathways through which honey exerts its antimicrobial effects, particularly in the context of resistant strains of bacteria. Furthermore, Zhang et al. [174], demonstrated that while hydrogen peroxide is a significant antimicrobial agent in various kinds of honey, the unique properties of Manuka honey, particularly its methylglyoxal (MGO) content, offer additional antimicrobial benefits that warrant further investigation.

More rigorous clinical trials and evidence-based guidelines are paramount in establishing honey’s role in modern medicine. Yaghoobi et al. [175] provided a comprehensive review of honey’s clinical applications in wound healing, emphasizing its antibacterial, anti-inflammatory, and antioxidant properties. However, the variability in honey’s composition based on floral source, processing, and storage conditions complicates its standardization for clinical use. This variability necessitates a systematic approach to clinical trials that assess honey’s efficacy in wound healing and establish standardized protocols for its application in clinical settings. Sherlock et al. [13] compared the antimicrobial activity of Ulmo honey and Manuka honey against common pathogens, revealing significant differences in their effectiveness. This further emphasizes the need for standardized testing protocols [13,165]. Innovations in honey-based pharmaceuticals and wound-care products are also on the horizon, driven by the increasing recognition of honey’s therapeutic properties. Mcloone et al. [176] highlighted the potential of honey combination therapies in treating skin and wound infections, suggesting that integrating honey with other antimicrobial agents could enhance its efficacy. This approach is particularly relevant in biofilm-associated infections, where traditional antibiotics often fail. The development of medical-grade honey formulations that combine honey with other active ingredients could provide a dual-action therapeutic strategy, enhancing both the antimicrobial activity and the ease of application. Pleeging et al. [177] demonstrated that supplemented medical-grade honey exhibited enhanced activity against *P. aeruginosa* biofilms, indicating a promising avenue for future research and product development.

Moreover, honey processing and its impact on antimicrobial activity is an area ripe for exploration. Chen et al. [178] investigated how standard heat and filtration processes affect the levels of hydrogen peroxide and overall antimicrobial activity in honey. Their findings suggest that while processing can alter the antimicrobial properties of honey, understanding these changes can lead to the development of more effective honey-based products. This is particularly important as the demand for natural and effective wound-care solutions continues to rise. Additionally, the work of Burlando and Cornara [179] emphasizes the growing interest in the dermatological applications of honey, advocating for more scientific investigations into its mechanisms of action and potential formulations for skin care. The antimicrobial properties of honey, particularly against biofilms, are critical in addressing the challenges posed by chronic wounds and resistant infections. Research by Lü et al. [112] demonstrated that honey could inhibit and eliminate biofilms produced by *P. aeruginosa*, a common pathogen in chronic wounds. This capability positions honey as a valuable adjunct in wound management strategies, particularly in cases where biofilm formation complicates treatment. Furthermore, as noted by Maillard et al. [180], the synergistic effects of honey with conventional antibiotics suggest that honey could play a crucial role in overcoming antibiotic resistance. The potential for honey to enhance the efficacy of existing antibiotics while simultaneously reducing the risk of resistance development presents a compelling case for its integration into modern therapeutic regimens. Honey delivery systems have been explored to optimize honey’s therapeutic efficacy. For instance, Hixon et al. [181] investigated the incorporation of Manuka honey into various tissue engineering scaffolds, including cryogels, hydrogels, and electrospun scaffolds. Their study assessed how different scaffold geometries affect bacterial clearance and adhesion. They found that electrospun scaffolds exhibited a faster release of honey, leading to more significant bacterial clearance, while cryogels and hydrogels provided extended-release profiles suitable for long-term applications. This research highlights the potential of tailored scaffold designs to enhance the antimicrobial properties of honey in medical applications.

## 11. Conclusions

Honey’s remarkable antimicrobial properties make it an asset in modern healthcare, particularly in combating antibiotic-resistant infections. Through a combination of mechanisms—such as hydrogen peroxide production, high sugar concentration, and bioactive compounds like phenols and bee defensin-1—honey demonstrates potent activity against many pathogens, including antibiotic-resistant strains like MRSA. The variability in its antimicrobial potency, influenced by its floral source and environmental conditions, underscores the importance of standardizing honey types for medical use. As antibiotic resistance continues to rise, honey offers a promising adjunct to conventional therapies in treating infections and enhancing the efficacy of existing antibiotics, making it a vital tool in addressing the growing public health challenge. Further research into honey’s specific bioactive components and their mechanisms will continue to shape its role in clinical settings. Studies have already shown that honey can disrupt bacterial biofilms, enhance wound healing, and reduce inflammation, making it an effective treatment for chronic wounds, especially in patients with compromised immune systems. As scientific understanding expands, honey’s integration into mainstream healthcare will increase, offering a natural, effective alternative to synthetic antimicrobial agents. In the face of rising antimicrobial resistance, honey’s multifaceted applications provide hope, paving the way for its continued exploration and potential as a cornerstone of modern medical practice.

## Data Availability

Not applicable.
